# Inhibition of Toll-Like Receptor 4 Signaling Mitigates Microvascular Loss but Not Fibrosis in a Model of Ischemic Acute Kidney Injury

**DOI:** 10.3390/ijms17050647

**Published:** 2016-04-29

**Authors:** Pierre C. Dagher, Takashi Hato, Henry E. Mang, Zoya Plotkin, Quentin V. Richardson, Michael Massad, Erik Mai, Sarah E. Kuehl, Paige Graham, Rakesh Kumar, Timothy A. Sutton

**Affiliations:** 1 Department of Medicine, Division of Nephrology, Indiana University School of Medicine, Indianapolis, IN 46202, USA; pdaghe2@iu.edu (P.C.D.); thato@iu.edu (T.H.); hmang@iu.edu (H.E.M.); zplotkin@iu.edu (Z.P.); qrichardson991@gmail.com (Q.V.R.); michaelpmassad@gmail.com (M.M.); Erik.m.mai@gmail.com (E.M.); kuehly@mac.com (S.E.K.); grahamp@purdue.edu (P.G.); kumaryd@gmail.com (R.K.); 2 Department of Medicine, Division of Nephrology, Indiana University School of Medicine, R2-202, 950 West Walnut Street, Indianapolis, IN 46202, USA

**Keywords:** toll-like receptor 4, acute kidney injury, ischemia-reperfusion, fibrosis

## Abstract

The development of chronic kidney disease (CKD) following an episode of acute kidney injury (AKI) is an increasingly recognized clinical problem. Inhibition of toll-like receptor 4 (TLR4) protects renal function in animal models of AKI and has become a viable therapeutic strategy in AKI. However, the impact of TLR4 inhibition on the chronic sequelae of AKI is unknown. Consequently, we examined the chronic effects of TLR4 inhibition in a model of ischemic AKI. Mice with a TLR4-deletion on a C57BL/6 background and wild-type (WT) background control mice (C57BL/6) were subjected to bilateral renal artery clamping for 19 min and reperfusion for up to 6 weeks. Despite the acute protective effect of TLR4 inhibition on renal function (serum creatinine 1.6 ± 0.4 mg/dL TLR4-deletion *vs.* 2.8 ± 0.3 mg/dL·WT) and rates of tubular apoptosis following ischemic AKI, we found no difference in neutrophil or macrophage infiltration. Furthermore, we observed significant protection from microvascular rarefaction at six weeks following injury with TLR4-deletion, but this did not alter development of fibrosis. In conclusion, we validate the acute protective effect of TLR4 signal inhibition in AKI but demonstrate that this protective effect does not mitigate the sequential fibrogenic response in this model of ischemic AKI.

## 1. Introduction

Acute kidney injury (AKI) is a commonly encountered clinical entity with significant acute morbidity and mortality [1,2,3,4,5,6]. In addition to the acute consequences associated with an episode of AKI, there is growing evidence that maladaptive repair following AKI ushers in the development of chronic kidney disease (CKD) in patients that survive an episode of AKI [7,8,9,10]. The mechanisms associated with dysfunctional repair following AKI are not well characterized, although the development of interstitial fibrosis is a common final signature.

Toll-like receptors (TLRs) are a superfamily of transmembrane receptors that detect their respective ligands through molecular pattern recognition and play a central role in mammalian innate immunity. However, TLRs are expressed in cells other than the traditional cellular constituents of the innate immune system. Moreover, endogenous non-microbial ligands known as damage-associated molecular patterns (DAMPs) that are released during tissue injury can activate TLRs. Consequently, TLRs may serve as important monitors of tissue damage and modulators of disease in a variety of conditions including kidney disease [11]. Toll-like receptor 4 (TLR4), the receptor for endotoxin, is known to be expressed on kidney tubular epithelial cells as well as kidney microvascular endothelial cells [12,13]. Importantly, it has been demonstrated that DAMP activation of TLR4 on both kidney tubular epithelial cells and kidney microvascular endothelial cells promotes acute tubular injury and kidney dysfunction in ischemic AKI [13,14,15,16].

Recent investigations have suggested that the role of TLR4 activation extends beyond the acute phase of injury in various models of kidney disease. Indeed, TLR4 has been implicated in the modulation of kidney fibrosis and the development of chronic kidney disease (CKD) following acute injury. Inhibition of TLR4 signaling mitigates the fibrotic response in mouse models of folic acid nephropathy, 5/6th nephrectomy with concomitant continuous angiotensin infusion, and unilateral ureteral obstruction [17,18,19,20].

In this study, we examine the effects TLR4 signaling on the development of kidney fibrosis in a mouse model of ischemic AKI. Similar to previous studies, we find that the functional deletion of TLR4 provides acute protection following ischemic injury. In contrast, this acute protection did not translate into diminished fibrosis six weeks after injury despite a significant protection of microvascular rarefaction. These studies suggest that the role of TLR4 signaling may have disparate outcomes depending on the initial injurious event.

## 2. Results

### 2.1. Functional Deletion of TLR4 Confers Acute Protection in a Model of Ischemic AKI

**Figure 1:**
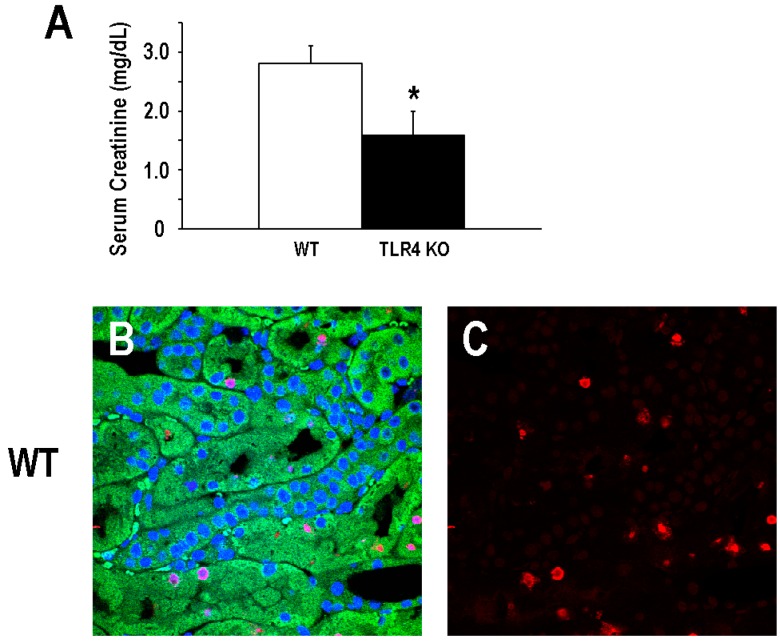

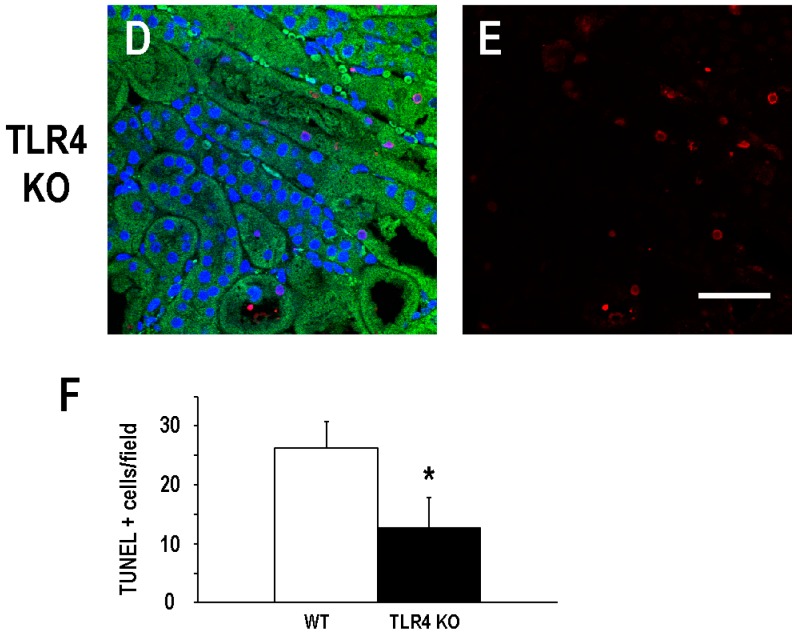
Deletion of TLR4 confers acute protection in a model of ischemic AKI. (**A**) Mean serum creatinine values (±SD) at 24 h following a 19-min bilateral renal artery clamp in background control (WT) and TLR4-deletion (TLR4 KO) mice. Representative images (60×) of TUNEL staining are shown for background control (WT; **B**,**C**) and TLR4-deletion (TLR4 KO; **D**,**E**) mice at 24 h following a 19-min bilateral renal artery clamp; Panels **B** and **D** are composite images of TUNEL (red), DAPI (blue), and tubular auto-fluorescence (green); Panels **C** and **E** are fields identical to the adjacent composite field demonstrating TUNEL staining alone. Quantitation of TUNEL positive tubular cells at 24 h is demonstrated in panel **F** (mean TUNEL-positive tubular cells/field ±SD; 5–7 fields/animal; *n* = 4, * *p* < 0.05). TUNEL-positive tubular cells were counted by a blinded observer. Scale bar = 50 µm.

As mentioned above, previous studies have demonstrated that the functional deletion of TLR4 confers protection in a murine model of ischemic AKI [13,14]. For our studies, we used a mouse with a spontaneous deletion in the *Tlr4* coding sequence such that no mRNA or protein is expressed (B6.B10ScN-*Tlr4*^lps−del^/JthJ) that has been backcrossed into C57BL/6 for at least five generations. Consistent with previous studies, we observed that B6.B10ScN-*Tlr4*^lps−del^/JthJ mice (henceforth called TLR4-deletion mice) were protected from injury in a model of ischemic AKI. Mean serum creatinine determined 24 h after a 19-min bilateral renal artery clamp was 1.6 ± 0.4 mg/dL in the TLR4-deletion mice as compared to 2.8 ± 0.3 mg/dL in C57BL/6 background mice (henceforth called WT mice; Figure 1A). In addition, TLR4-deletion mice demonstrated a significant reduction in tubular apoptosis, particularly proximal tubular cell apoptosis, as demonstrated by the mean TUNEL-positive tubular cells/field (12.8 ± 5.0/field) following ischemic kidney injury as compared to WT mice (26.2 ± 4.5/field; Figure 1B–F).

### 2.2. Functional Deletion of TLR4 Did Not Significantly Alter the Early Inflammatory Response in a Mouse Model of Ischemic AKI

**Figure 2:**
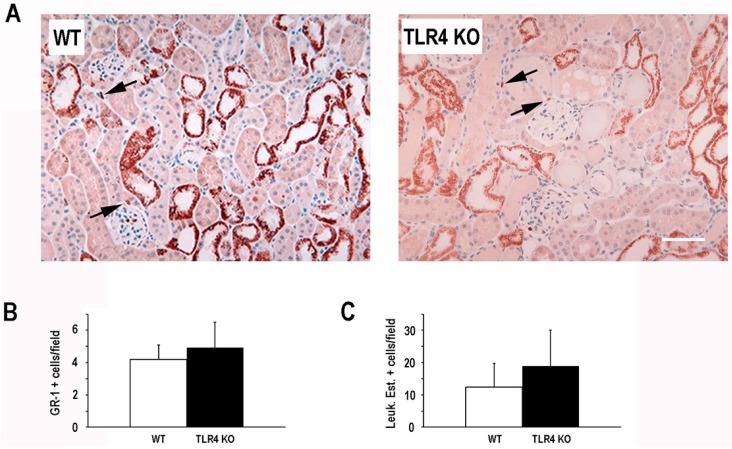

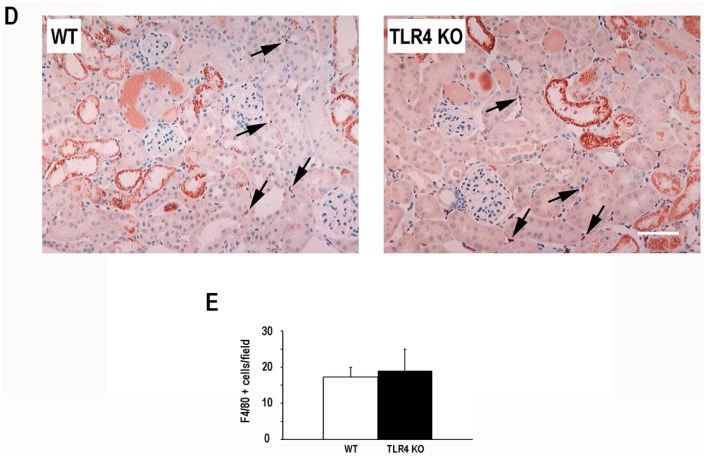
Deletion of TLR4 did not significantly alter the early inflammatory response in a mouse model of ischemic AKI. Panel **A** demonstrates representative Gr-1 staining (brown, indicated by arrows) for WT and TLR4-deletion (TLR4 KO) mice at 24 h following a 19-min bilateral renal artery clamp. Hematoxylin (blue) was used as a counterstain. Non-specific staining of some tubules was noted; Panels **B** and **C** show quantitation of nucleated, non-tubular Gr-1-positive cells/field and leukocyte esterase-positive cells/field (images not shown) in kidneys from the two groups at 24 h following a 19-min bilateral renal artery clamp (mean Gr-1+ or leukocyte esterase+ nucleated, non-tubular cells/field ±SD; 5–7 fields/animal; *n* = 4); Panel **D** demonstrates representative F4/80 staining (brown, indicated by arrows) for WT and TLR4-deletion (TLR4 KO) mice at 24 h following a 19-min bilateral renal artery clamp. Hematoxylin (blue) was used as a counterstain. Non-specific staining of some tubules was noted; Panel **E** shows quantitation of nucleated, non-tubular F4/80-positive cells/field in kidneys from the two groups at 24 h following a 19-min bilateral renal artery clamp (mean F4/80+ nucleated, non-tubular cells/field ±SD; 5–7 fields/animal; *n* = 4). Scale bar = 40 µm.

In view of the role that TLR4 plays in activating the innate inflammatory response and the role that inflammation plays in the pathogenesis of ischemic AKI [21], we next examined the number of resident tissue inflammatory cells following ischemic injury. Interestingly, we did not observe a significant difference in the number of neutrophils (GR-1 positive cells, leukocyte esterase positive cells) or macrophages (F4/80 positive cells) in TLR4-deletion mice compared to WT mice 24 h after ischemic injury (Figure 2).

### 2.3. Functional Deletion of TLR4 Preserved Microvascular Density Following an Episode of Ischemic AKI

**Figure 3:**
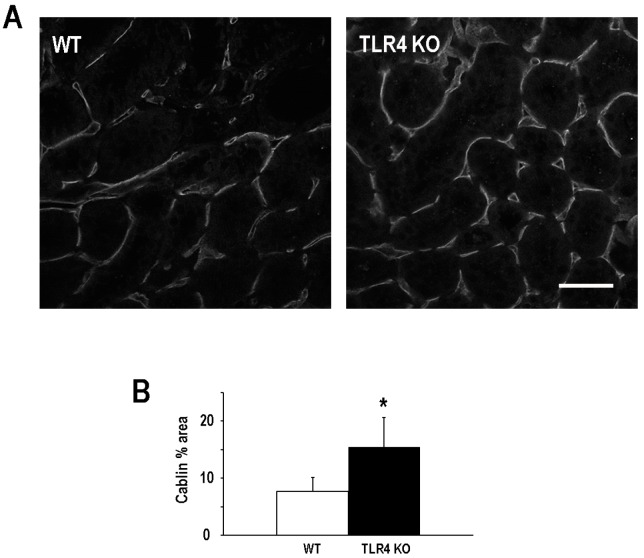
Deletion of TLR4 preserved microvascular density following an episode of ischemic AKI*.* Panel (**A**) demonstrates representative images of cablin immunostaining for WT and TLR4-deletion (TLR4 KO) mice at six weeks following a 19-min bilateral renal artery clamp; Panel (**B**) shows quantitation of cablin immunostaining (percent total area staining positive for cablin/field ±SD; 5–7 fields/animal; *n* = 4; * *p* < 0.05). Scale bar = 50 µm.

Endothelial injury and the loss of microvascular density following an episode of ischemic AKI [22,23,24,25] has been associated with persistent renal hypoxia [26] that can usher in the development and the progression of AKI to CKD [22,27]. TLR4 activation on kidney microvascular endothelial cells contributes to injury and the overall decrement in function during ischemic AKI [16]. Consequently, we next evaluated the impact of TLR4 deletion on microvascualr density six weeks after an episode of ischemic AKI. Microvascular density, as determined by % area of cablin immunostaining [25,28], was better preserved after ischemic injury in the TLR4-deletion mice (16.9% ± 5.2%) as compared to the WT mice (7.7% ± 2.4%; Figure 3).

### 2.4. Functional Deletion of TLR4 Did Not Significantly Alter the Development of Fibrosis Following an Episode of Ischemic AKI

**Figure 4:**
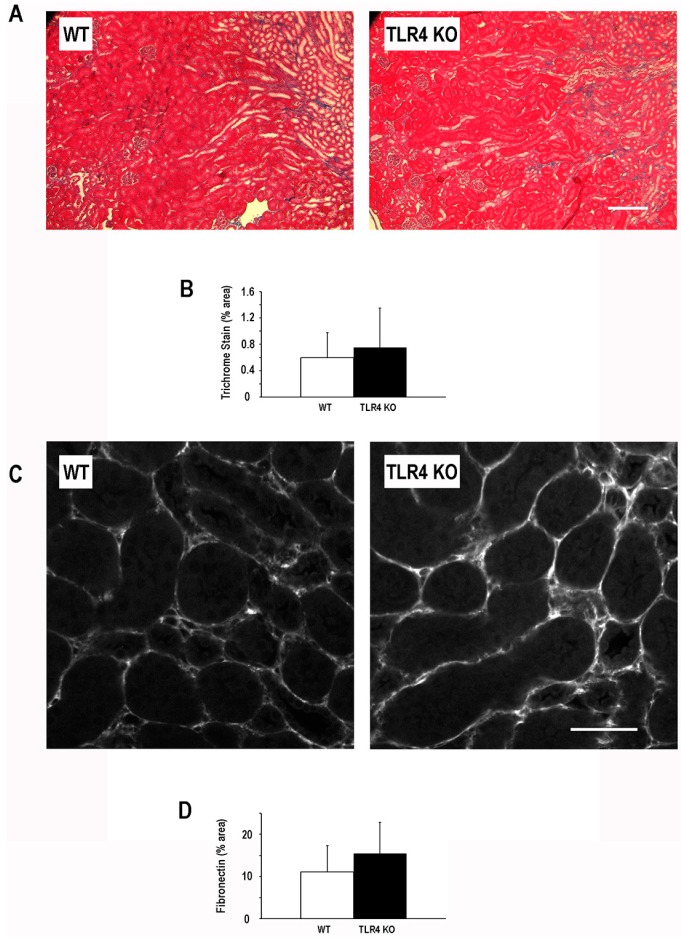
Deletion of TLR4 did not significantly alter the development of fibrosis following an episode of ischemic AKI. Panel (**A**) demonstrates representative trichrome images for WT and TLR4-deletion (TLR4 KO) mice at six weeks following a 19-min bilateral renal artery clamp; scale bar = 100 µm; Panel (**B**) shows quantitation of collagen/fibrosis (blue) on trichrome stained images (percent total area staining blue/field ±SD; 5–7 fields/animal; *n* = 4); Panel (**C**) demonstrates representative images of fibronectin immunostaining for WT and TLR4-deletion (TLR4 KO) mice at six weeks following a 19-min bilateral renal artery clamp; Scale bar = 50 µm; Panel (**D**) shows quantitation of fibronectin immunostaining on (percent total area staining positive for fibronectin/field ±SD; 5–7 fields/animal; *n* = 4).

To further evaluate the consequence of TLR4-deletion on chronic sequelae of ischemic AKI, we examined the fibrogenic response at six weeks following injury. Despite the acute protective effect of TLR4-deletion in this model of ischemic AKI and the mitigation of microvascular loss six weeks following injury, the degree of fibrosis at six weeks following injury as determined by Masson’s trichrome was not significantly different (Figure 4) between TLR4-deletion mice (0.75% ± 0.60%) and WT mice (0.60% ± 0.38%). In addition, fibronectin an extracellular matrix protein that can serve as a nidus for fibrosis in the kidney [29,30,31,32] was not significantly different six weeks after injury in the TLR4-deletion mice (15.4% ± 7.4%) as compared to the WT mice (11.1% ± 6.2%; Figure 4).

## 3. Discussion

Several studies have substantiated that activation of TLR4 signaling promotes acute injury in ischemic AKI [13,14,16,33]. Studies in animal models of kidney transplant-associated ischemia-reperfusion injury and human kidney transplant-associated delayed graft function have further supported this finding [34]. Our findings in this study further validate the importance of TLR4 signaling in ischemic AKI. Similar to previous studies, we demonstrate that TLR4-deletion mitigates functional impairment (serum creatinine) and tubular apoptosis following acute ischemic kidney injury. Interestingly, despite the protective effect of TLR-deletion, we did not observe a significant decrease in either neutrophil or macrophage accumulation at 24 h following injury. This observation contrasts with prior studies of TLR4-deletion in a mouse model of ischemic AKI [13,14]. Only nucleated interstitial cells that had immunostaining with the antibody to identify neutrophils or macrophages were counted in our study, whereas all cells staining positive with the selected antibody were counted in prior studies. This distinction in quantitative approach, in light of the apparent nonspecific tubular epithelial cell staining of each antibody demonstrated by us and in previous studies, may partially explain the difference in findings for both neutrophil and macrophage accumulation. In addition, the kinetics of macrophage infiltration from prior studies demonstrate a difference between TLR4-deficient and WT mice that is smallest at 24 h, the time point examined in our study, and largest at five days after injury [13]. Accordingly, our findings in general underscore the significance of early, non-inflammatory cell TLR4 activation in the pathophysiology of AKI demonstrated by others [13,35,36] and suggest that the cellular context of TLR4 activation may be an important determinant of outcome following a particular insult.

Given the acute protective effect of TLR4-deletion that we observed in ischemic AKI, it was not surprising to find that microvascular integrity was better maintained following injury in the TLR4-deficient mice. However, our finding that acute protection and diminished microvascular rarefaction did not translate into reduced fibrosis was unanticipated provided chronic hypoxia is believed to be one of the drivers for kidney fibrosis [37,38]. Inhibition of TLR4 signaling has generally been shown to attenuate fibrosis in rodent models of fibrotic kidney disease [17,18,20], although this is not a universal finding [39]. Adding to this complexity is that, in at least one study, fibrosis was diminished despite enhanced tubular injury [17], contrary to general intuition. Our observation that tubular injury was attenuated without altering long-term fibrosis may be a reflection of the fact that minimal fibrosis occurred with this model as compared to the more fibrotic models such as UUO. Giving credence to this notion is the finding in a prior study that despite the increase in fibrosis following 5/6ths nephrectomy, there was no difference in fibrosis between WT and TLR4-deficient mice [18]. Only after adding the chronic infusion of angiotensin II to the 5/6ths nephrectomy model as an additional provocative measure to increase the overall fibrogenic response was a difference in fibrosis observed between WT and TLR4-deficient mice.

Of note, we have previously reported discordance between microvascular rarefaction and fibrosis [23,40]. The observation of this discordance may not be unexpected given the number of posited mechanisms leading to increased kidney fibrosis following injury [41,42]. Furthermore, plausible mechanisms for how TLR4 activation contributes to kidney fibrosis have support in the literature, and most evoke some aspect of inflammation. Consequently, since we did not observe any difference in the acute cellular inflammatory response between TLR4-deletion mice and WT mice in our model, this may partially explain why we did not observe differences in fibrosis. Lastly, variation in the susceptibility to fibrosis of background strains in these models may be a more mundane explanation to the lack of difference in fibrosis observed. Germane to this point is that many of the studies examined TLR4-deficiency on a C3H background, whereas this study as well as another study [39], demonstrating no difference in fibrosis, utilized a mouse with TLR4-deficiency on a C57BL/6 background.

In conclusion, we demonstrate that TLR4-deficiency diminishes acute tubular injury and organ dysfunction without significantly modifying inflammatory cell infiltration in an animal model of ischemic AKI. This acute protection is associated with preserved microvascular integrity but does not alter fibrosis at six weeks after injury. The outcome of TLR4 inhibition following acute ischemic injury to the kidney may be dependent on the context and cellular origin of TLR4 activation and further work is needed to clarify the benefit of TLR inhibition on the chronic outcomes of ischemic AKI.

## 4. Experimental Section

### 4.1. Animals and Experimental Models

All animal protocols were approved by the Indiana University Institutional Animal Care Committee and conform to the NIH Guide for the Care and Use of Laboratory Animals. B6.B10ScN-*Tlr4*^lps−del^/JthJ (TLR4-deficient) and C57BL/6 (WT background control) male mice weighing 20–25 grams (10–12 weeks) were obtained from Jackson Laboratory (Bar Harbor, Maine, ME, USA). Bilateral renal artery clamp surgery was performed following induction of anesthesia with 5% isoflurane and maintenance of anesthesia with 1.5% isoflurane and buprenorphine HCL (0.01 mg/kg) subcutaneously. Core body temperature (37 °C) of the mice was maintained by placement on a homeothermic surgical pad thoughout the surgical procedure. The renal pedicles were isolated through a midline incision, and bilateral renal ischemia was induced by clamping the renal pedicles for 19 min with microaneurysm clamps. Reperfusion of the kidneys was monitored visually following removal of the microaneurysm clamps. Five hundred microliters of prewarmed (37 °C) sterile saline was instilled into the peritoneum just prior to closing the abdominal wound. Animals recovered on a homeothermic surgical pad until the righting reflex was restored. An identical procedure was followed with the exception of immediate release of the microaneurysm clamps in sham animals. Reperfusion time was 24 h prior to euthanasia for the examination of acute tissue changes or six weeks prior to euthanasia for the examination of chronic tissue changes . Kidneys were harvested at the time of euthanasia and subsequently processed for histopathology studies as described elsewhere in this section. Measurement of serum creatinine concentrations was performed at the University of Texas Southwestern O’Brien Kidney Research Core Center, Dallas, TX, USA.

### 4.2. Histopathology and Microscopy

Kidneys were rapidly perfused fixed with either 100% methanol (cablin staining only) or 4% paraformaldehyde at the time of euthanasia Kidney tissues were subsequently processed for immunostaining or standard histochemistry. Immunostaining was performed with primary antibodies to cablin (rabbit anti-human polyclonal IgG) [43], fibronectin (rabbit anti-rat polyclonal IgG; MD Biosciences, St. Paul, MN, USA), Gr-1 (NIMP-R14; Abcam, Cambridge, MA, USA), or F4/80 (Clone CI:A3-1; AbD Serotec, Raleigh, NC, USA). Secondary antibodies conjugated with Texas Red or horseradish peroxidase were purchased from Jackson Immunoresearch Laboratories, West Grove, PA, USA. Kidney tissue sections from sham animals and animals undergoing renal ischemia were incubated with secondary antibodies in the absence of primary antibodies as negative controls. Staining for leukocyte esterase was performed using a naphthol AS-D chloroacetate (specific esterase) kit (Sigma-Aldrich, St. Louis, MO, USA) on paraffin-embedded tissue as per the manufacturer’s instructions. Trichrome staining was performed using standard histochemistry procedures by the Indiana University Histopathology Lab.

Confocal immunofluorescent images of kidney tissue sections were collected at 60× magnification using a LSM-510 Zeiss confocal microscope (Peabody, MA, USA) or Olympus FV1000 confocal microscope (Center Valley, PA, USA). Trichrome-stained images and immunohistochemistry images of kidney tissue sections were obtained at 10× and 40× magnification respectively with a Nikon Microphot-SA equipped with SPOT RT Slider camera (Diagnostic Instruments. Inc.; Melville, NY, USA). Metamorph software (Universal Imaging, West Chester, PA, USA) was used to determine the percent of total area staining positive for fibrosis (trichrome), cablin, or fibronectin. Cells staining positive for TUNEL and nucleated interstitial cells staining positve for F4/80, GR-1, and leukocyte esterase in each field were manually counted. Tissue scoring and tissue cell counts were quantified by a blinded individual. Five to seven images were collected of a kidney from each animal.

### 4.3. Statistical Analysis

Data were analyzed for statistical significance using ANOVA and pairwise *t*-tests or a nested ANOVA for nested data within a study subject. All data are reported as means with SD. Significance was set at *p* < 0.05.
